# The Mechanism of Compound Anshen Essential Oil in the Treatment of Insomnia Was Examined by Network Pharmacology

**DOI:** 10.1155/2019/9241403

**Published:** 2019-06-04

**Authors:** Guilin Ren, Yu Zhong, Gang Ke, Xiaoli Liu, Huiting Li, Xiaofang Li, Qin Zheng, Ming Yang

**Affiliations:** ^1^Key Laboratory Breeding Base of Systematic Research and Utilization on Chinese Material Medical Resources Co-Founded by Sichuan Province and Ministry of Science and Technology, Pharmacy College, Chengdu University of Traditional Chinese Medicine, Chengdu 611137, China; ^2^Key Laboratory of Modern Preparation of Traditional Chinese Medicine, Ministry of Education, State Key Lab. of Innovation Drug and Effcient Energy-Saving Pharmaceutical Equipment, Jiangxi University of Traditional Chinese Medicine, Nanchang 330004, China; ^3^Luzhou People's Hospital, Luzhou 646100, China

## Abstract

The active component-target network and protein-protein interaction network of Compound Anshen essential oil were constructed. The target functions and related pathways were analyzed to explore the mechanism of Compound Anshen essential oil in the treatment of insomnia. GC-MS was used to detect the chemical composition of Compound Anshen essential oil, and the TCMSP, STITCH, TTD, and DrugBank databases were searched to predict and screen the targets of Compound Anshen essential oil in the treatment of insomnia. Cytoscape software was used to construct the network diagrams of the active component-action target and protein-protein interaction networks, ClueGO software was used to analyze the GO enrichment and KEGG pathway of the target, and the systemsDock website database was used for molecular docking. The analysis of the network results showed that the activity of Compound Anshen essential oil mainly involves biological processes such as the phospholipase C-activating G protein-coupled receptor signaling pathway, response to ammonium ions, calcium ion transport into the cytosol, and chloride transport. The results of molecular docking showed that linalool, caryophyllene, dibutyl phthalate, (-)-4-terpineol, and (-)-*α*-terpineol have good binding activity with ADRB2, DRD2, ESR1, KCNH2, NR1H4, NR1I2, NR1I3, and TRPV1 targets. This study demonstrates the multicomponent, multitarget, and multichannel characteristics of Compound Anshen essential oil and provides a new therapeutic idea and method for further research on the mechanism of Compound Anshen essential oil in the treatment of insomnia.

## 1. Introduction

Sleep is an important component of people's daily lives, and sleep time accounts for more than 30% of people's lives. However, as a result of increases in mental stress and other pressures, sleep disorder problems are becoming increasingly prevalent. Approximately 27% of people worldwide suffer from sleep disorders. Insomnia refers to a subjective experience of the patient not being satisfied with sleep time and/or quality, and insomnia affects the ability of an individual to function in society during the day [1, 2]. Insomnia is clinically manifested as sleep process disorder, daytime cognitive dysfunction, limbic system, and peripheral nerve dysfunction, all of which not only cause anxiety, depression, or fear but also lead to decreased mental activity and efficiency and an increased incidence of cardiovascular and mental diseases [3–5].

Currently, there are two main types of insomnia treatment methods: nondrug treatments, including health education, light therapy, and psychological and cognitive behavioral therapy. The second type of insomnia treatment is drug therapy, including benzodiazepines, nonbenzodiazepines, and antianxiety and antidepressant drugs. The main efficacy of these standard drugs for insomnia is to help improve patients' symptoms, such as difficulty falling asleep and difficulty maintaining sleep. However, due to insufficient selectivity of targeted receptors and inhibition of the central nervous system, prolonged use of these standard drugs will lead to dependence and tolerance, side effects such as sleepiness and hangovers in the morning, and various adverse reactions including withdrawal symptoms that occur once drug administration is stopped [6].

An increasing number of researchers are keen to explore new, simple, and safe auxiliary or alternative solutions to treat insomnia. Aromatherapy is an alternative therapy to assist in medical treatment by using the pure essential oils of an aromatic plant. People extract essential oils with different smells and colors from naturally aromatic plants. These essential oils mainly contain terpenes, aldehydes, esters, alcohols, and other chemical components. Essential oils can be directly inhaled, bathed in, or used in massages, and these essential oils have been used to reduce insomnia, anxiety, pain, fatigue, and other symptoms [7–10].

Compound Anshen essential oil can cure insomnia by olfaction. Compound Anshen consists of seven essential oils, including sandalwood essential oil, aloe essential oil, rose essential oil, and lavender essential oil, Frankincense essential oil, neroli essential oil, and sweet orange essential oil. A large number of studies have found that olfaction is closely related to insomnia. The olfactory pathway not only transmits the sense of smell but also regulates advanced functions of the brain, such as learning, memory, emotion, visceral activity, alertness, and sleep. Olfactory transmission of information occurs through the neurotransmitters glutamate (Glu), gamma-aminobutyric acid (GABA), 5-hydroxytryptamine (5-HT), dopamine (DA), and norepinephrine (NA), and these transmitters and insomnia pathogenesis are closely related [11, 12]. At present, the efficacy of Compound Anshen essential oil has been verified by pharmacodynamics, and a patent has been applied for. However, the mechanisms, signaling pathways, and material bases of the action of Compound Anshen essential oil related to insomnia are unclear. Network pharmacology builds a “drug-target-pathway” multilevel network based on the biological molecular network to analyze the pharmacodynamically active ingredients and the possible molecular network mechanisms, aimed at establishing the synergistic relationship between the multicomponents, multichannels, and multitargets of traditional Chinese medicine and its compounds [13–16].

In this paper, the mechanism of Compound Anshen essential oil in the treatment of insomnia was studied by network pharmacology. The purpose of this study was to explore the effect of essential oil on physiological mechanisms and signaling pathways involved in insomnia by using network pharmacology, drug-targeted interaction databases, and biological analysis methods. Therefore, clinical research on the anti-insomnia effect mediated by Compound Anshen essential oil could be implemented on this basis.

## 2. Materials and Methods

### 2.1. Determination of Chemical Components and Screening of Targets

The chemical composition of Compound Anshen essential oil was determined by gas chromatography-mass spectrometry (GC-MS). Searches through the PubChem (https://pubchem.ncbi.nlm.nih.gov/), STITCH (http://stitch.embl.de/), and TCMSP (http://lsp.nwu.edu.cn/tcmsp.php) databases provided chemical composition targets.

### 2.2. Screening of Potential Targets for Insomnia

The TTD (https://db.idrblab.org/ttd/), DrugBank (https://www.drugbank.ca/), and DisGeNET(http://www.disgenet.org/web/DisGeNET/menu/home) databases were searched to identify targets related to insomnia.

### 2.3. Construction of Active Component-Target Network

The intersection of the chemical target and insomnia disease target was determined and taken as the potential target, and the components of the chemical composition corresponding to the potential target were taken as the potential active component. The UniProt database (http://www.uniprot.org/) was used to correct all target names that were subsequently retrieved as official symbols to obtain the target and UniProt number related to the active components. The active components and targets of Compound Anshen essential oil were introduced into Cytoscape 3.7.0 software to construct the network of the Compound Anshen essential oil active component-action target and to predict the main targets and chemical components of Compound Anshen essential oil that are related to treating insomnia.

### 2.4. Construction and Analysis of the Interaction Network of Action Targets

The STRING database (https://string-db.org/) is a database containing known and predicted protein-protein interactions (PPI), and there have been a large number of protein-protein interactions collected, including both experimentally verified data and predicted data using bioinformatics methods. The action target was imported into the STRING database, the species was defined as human, the interaction relationship was obtained, and the result was saved in the RTF format, which retained the node (node 1, node 2) and combined rate score (combined score) information. This information was imported into the Cytoscape 3.7.0 software where the interaction network was drawn and analyzed, with analysis results saved. The node size and color settings were used to reflect the number of combined targets (degree) of the components using Cytoscape tools.

### 2.5. Gene Ontology and Pathway Enrichment Analysis

The biological process, molecular function, cell component, and KEGG pathway enrichment analyses of the action target of the chemical composition were applied by ClueGO to further reveal the mechanism of Compound Anshen essential oil.

### 2.6. The Main Active Ingredient—Target Molecular Docking

The main chemical components in Compound Anshen essential oil were selected and interactions verified by molecular docking between the main pharmacodynamic components and the protein using the systemsDock website database. The protein name and 3D structure of the compound were uploaded, and the docking analysis was conducted.

## 3. Results and Discussion

### 3.1. Determination of the Chemical Components in Compound Anshen Essential Oil

**Figure 1:**
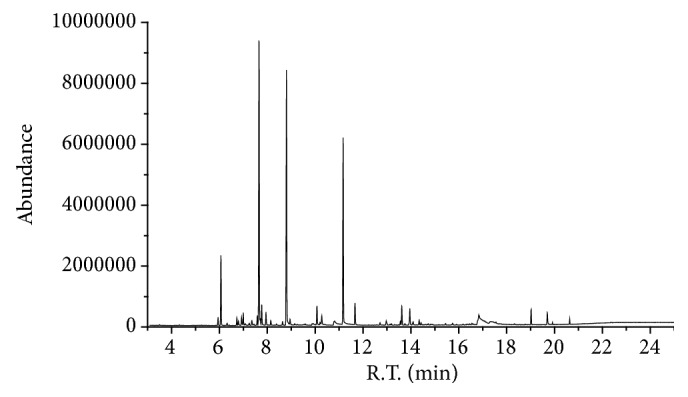
GC-MS chromatogram of Compound Anshen essential oil.

In this paper, the chemical components of Compound Anshen essential oil were determined by gas chromatography-mass spectrometry (GC-MS) (Figure 1). Gas chromatographic conditions: Agilent DB-624 (30m x 320*μ*m x 1.8*μ*m) capillary column was used, the carrier gas was high purity He (99.999%), the sample volume was 1*μ*L, the shunt ratio was 40:1, and the flow rate was 1mL/min. Temperature program: initial temperature 40°C (1 min), with 10°C/min up to 220°C, and then in 25°C/min up to 280°C (keep 9 min). Mass spectrometry conditions: EI ion source, electron energy 70 e V, ion source temperature 230°C, MS quadrupole temperature 150°C; Interface temperature 250°C, solvent 3.0 min delay, quality scan pattern full scan, scan range of 30 ~ 650 amu. Finally 22 essential oil chemical components were identified by GC-MS (Table 1). Linalool, D-limonene, and linalyl anthranilate are the three ingredients with the highest content, accounting for more than 60% of the total composition. Studies have shown that linalool has sedative and hypnotic effects. Linalool is a major component of lavender and may be used subcutaneously, peritoneally, orally, or inhaled [17–19]. Studies have shown that linalool has a significant anxiolytic effect. Inhalation of linalool can lead to sedation in mice, increase the sleep time induced by pentobarbital, reduce spontaneous behavior, and lower body temperature without affecting motor coordination [20–23]. D-limonene has been found to have a central nervous sedative effect [24]. Linalyl anthranilate has been found to have a sedative effect [25, 26].

### 3.2. Retrieval of Potential Targets

The protein targets of Compound Anshen essential oil were searched via the TCMSP, STITCH, and PubChem databases for each chemical component. Through the TTD, DrugBank, and DisGeNET databases, the retrieved results were integrated to obtain the insomnia-related disease protein targets. A total of 39 potential targets (Table 2) were obtained based on the intersection of protein targets acting on chemical components and those related to insomnia. Neurophysiological studies have proven that sleep is an active and rhythmic process in the central nervous system. Many of these neurotransmitters are involved in this process and play a key role in helping regulate sleep. GABA is an important inhibitory neurotransmitter in the central nervous system that can inhibit activated neurons and has a protective effect on neurons. Many studies have shown that GABA abnormalities are closely related to insomnia [27, 28].

### 3.3. Network Construction of Active Component-Action Target

**Figure 2:**
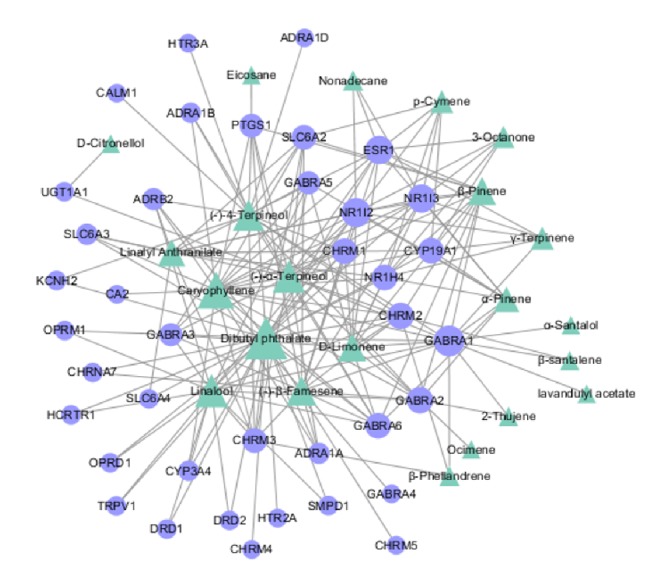
Component-target network of Compound Anshen essential oil. The component-target network was built by the potential targets and active components. Targets (blue round nodes) were connected to components (green triangles).

Cytoscape software was used to construct the network of chemical composition and targets of action (Figure 2), which has 61 nodes. The blue round nodes represent the targets of Compound Anshen essential oil, and the green triangle nodes represent the chemical composition of Compound Anshen essential oil. The “degree” represents the action intensity, and the higher the degree value is, the larger the node is. Each edge represents the interaction between the active component and the target. The results showed that the targets GABRA1, NR1I2, ESR1, NR1I3, GABRA2, and CHRM2 were highly correlated with the chemical components, among which GABRA1 was associated with 15 chemical components, NR1I2 was associated with 13 chemical components, and ESR1 and NR1I3 were associated with 12 chemical components.

GABA, the major inhibitory neurotransmitter in the vertebrate brain, mediates neuronal inhibition by binding to the GABA/benzodiazepine receptor and opening an integral chloride channel. GABA is an inhibitory neurotransmitter that is widely distributed throughout the central nervous system. GABA can inhibit neurons in the nervous system, has a neuroprotective effect, and can function as a hypnotic, sedative, and anxiolytic among other functions. If Glu is released in excess, excitatory neurotoxicity will be generated, which will induce insomnia. The inhibitory neurotransmitter GABA can inhibit and protect the central nervous system, which promotes sleep [29]. The muscarinic acetylcholine receptor mediates various cellular responses, including inhibition of adenylyl cyclase, breakdown of phosphoinositides and modulation of potassium channels, through the action of G proteins. The primary transducing effect is phosphoinositol (PI) turnover [30, 31].

### 3.4. Construction and Analysis of the Interaction Network of Action Targets

**Figure 3:**
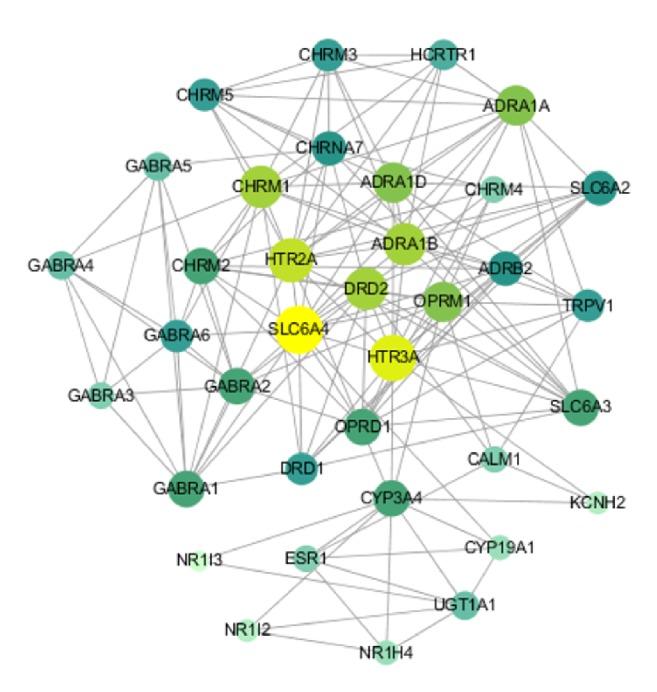
Protein-protein interaction network for Compound Anshen essential oil. The size and color of the nodes represent the value of the degree.

The network of protein-protein interactions (PPIN) (Figure 3) was constructed through the STRING database and Cytoscape software. The edges represent the association between a pair of action targets, the nodes represent the action target, and the “degree” value represents its action intensity. The higher the degree value is, the larger the node is, and the higher degree values are indicated by the color changes from light green to yellow.

According to the calculation based on the STRING database, the combination ability of DRD2 and SLC6A3 was the strongest, and the combined score values reached 0.99. According to the target interaction network diagram, SLC4A4 is in the center of the target, and its degree value is the largest, followed by HTR3A, HTR2A, DRD2, OPRM1, ADRA1B, ADRA1D, CHRM1, and ADRA1A.

The sodium-dependent serotonin transporter terminates the action of dopamine through its high affinity sodium-dependent reuptake into the presynaptic terminal, and dopamine can be regulated in cognitive-motor and other behaviors related to arousal. HTR2A and HTR3A are two of the several different 5-hydroxytryptamine (serotonin) receptors. Serotonin neurons can project fibers along the midline of the brainstem to the basal forebrain, thalamus, hypothalamus, and other regions, affecting sleep awakening and eye opening. Serotonin promotes arousal and inhibits rapid-eye-movement (REM) sleep [32, 33].

### 3.5. Gene Ontology and Pathway Enrichment Analysis

The ClueGO database connects the GO database to Cytoscape, which is an important plug-in for the Cytoscape visual analysis software. ClueGO analysis technology enables the use of similar descriptors for the function of target products from different databases and then classifies and analyzes the targets from biological processes, molecular functions, and cellular components, as well as the enrichment analysis of KEGG pathway (P≤0.05), to predict the mechanism of Compound Anshen essential oil in the treatment of insomnia.

**Figure 4:**
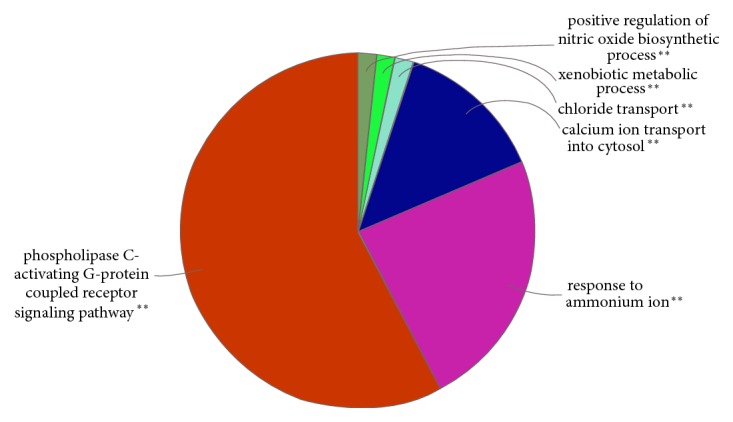
Enriched gene ontology terms for biological process (BP) of potential targets of the main active ingredients of Compound Anshen essential oil.

The retrieved protein targets of Compound Anshen essential oil were examined through ClueGO-mediated enrichment analysis by employing GO terms for the annotation of the biological functions. These GO terms were classified into groups. These groups were mainly involved in responses to the phospholipase C-activating G protein-coupled receptor signaling pathway, responses to ammonium ions, calcium ion transport into the cytosol, chloride transport, etc. (Figure 4). These observations are of great significance for further understanding the mechanisms of Compound Anshen essential oil.

**Figure 5:**
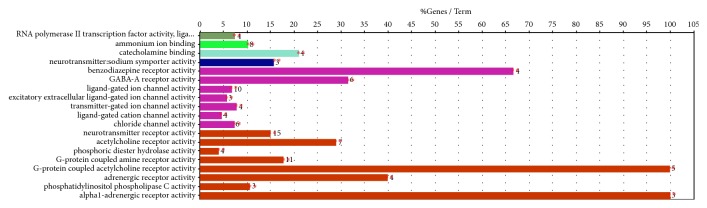
Enriched gene ontology terms for molecular function (MF) of potential targets of the main active ingredients of Compound Anshen essential oil.

The targets of molecular function (Figure 5) mainly involved neurotransmitter receptor activity, ligand-gated ion channel activity, G protein-coupled amine receptor activity, ligand-gated ion channel activity, ammonium ion binding, acetylcholine receptor activity, and many genes related to the molecular functions described above.

**Figure 6:**
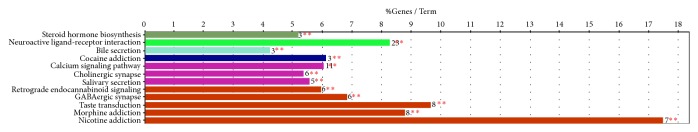
Enriched KEGG pathway of potential targets of the main active ingredients of Compound Anshen essential oil.

The cellular component targets are related only to the GABA-A receptor complex, postsynaptic cell membrane, and dendritic membrane. The enrichment analysis of the KEGG pathway (Figure 6) showed that these action targets were mainly related to neuroactive ligand-receptor interactions, calcium signaling pathways, taste transduction, morphine addiction, GABAergic synapses, etc. It has been noted that aloe essential oil has sedative and hypnotic effects, and its mechanism may be related to the regulation of the GABAergic system [34].

### 3.6. The Main Active Ingredient—Target Molecular Docking

Linalool, caryophyllene, dibutyl phthalate, (-)-4-terpineol, and (-)-*α*-terpineol are the most important active ingredients which have the highest degree in Compound Anshen essential oil. The systemsDock website database was used to verify the molecular docking of the main medicinal ingredients and proteins. In brief, the systemsDock website database was logged into; the target name was uploaded, which included linalool, caryophyllene, dibutyl phthalate, (-)-4-terpineol, and (-)-*α*-terpineol in.sdf format; and the 3D structure and docking were analyzed.

**Figure 7:**
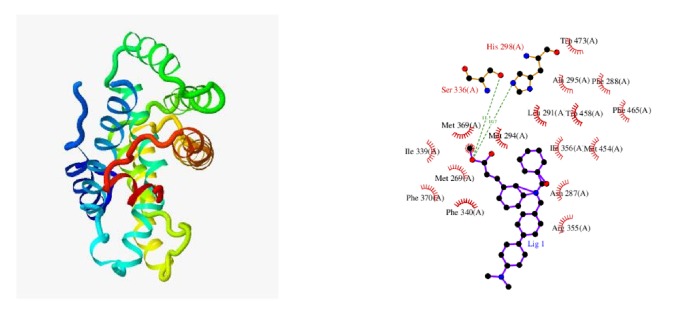
Docking diagram of NR1H4 and caryophyllene, and the 2D protein-ligand interaction.

It is generally considered that a docking score greater than 4.25 indicates a certain binding activity between the docking molecule and the target, values greater than 5.0 indicate good binding activity between the docking molecule and the target, and values greater than 7.0 indicate strong binding activity. The docking results showed that the average docking score of the chemical components, i.e., linalool, caryophyllene, dibutyl phthalate, (-)-4-terpineol, and (-)-*α*-terpineol, with the targets, i.e., ADRB2, DRD2, ESR1, KCNH2, NR1H4, NR1I2, NR1I3, and TRPV1, was 5.36. Caryophyllene and NR1H4 have a docking score of 7.335 (Figure 7). Caryophyllene and NR1I3 have a docking score of 7.249. This analysis showed that linalool, caryophyllene, dibutyl phthalate, (-)-4-terpineol, and (-)-*α*-terpineol have good binding activity with ADRB2, DRD2, ESR1, KCNH2, NR1H4, NR1I2, NR1I3, and TRPV1 targets, and the analysis showed that caryophyllene has strong binding activity with NR1H4 and NR1I3.

## 4. Conclusions

Compound Anshen essential oil has a sedative and tranquilizing effect and can be used to treat insomnia. The prescription consists of 7 essential oils, including sandalwood, aloe, rose, and lavender essential oils. The targets of the main active components of Compound Anshen essential oil are distributed in different pathways, and the mutual regulation of multiple components and targets is a possible mechanism of action in the treatment of insomnia.

From the component-target network diagram, we determined the five chemical components with the highest degree. We docked those five chemical components with the highest degree to the target, and NR1I2, NR1I3, and ESR1, which are the targets with high docking scores, are also the targets with a large degree in the component-target network diagram. This result indicates that Compound Anshen essential oil is closely related to the targets NR1I2, NR1I3, and ESR1 in the treatment of insomnia. However, there are few studies on the relationship between NR1I2, NR1I3, and ESR1 and sleep. Therefore, understanding how Compound Anshen essential oil regulates sleep through these targets is worthy of further study. Linalool, a compound with a high degree value, is also a component with a high content level, which indicates that linalool plays a vital role in the treatment of insomnia with Compound Anshen essential oil.

In summary, the network pharmacology results showed that the active components in the essential oil of the Anshen recipe acted on targets involved in a variety of biological processes, molecular functions, and cellular components, which reflected the characteristics of the multicomponent, multitarget, and multipathway action of the essential oil of Compound Anshen. In this paper, the active component-target network diagram showed the characteristics of Compound Anshen essential oil in the treatment of insomnia. The protein interaction network predicted the important target proteins of Compound Anshen essential oil for insomnia. There were mutual relations between the targets, which was a complex interaction network rather than a simple, single-target interaction. GO enrichment pathway analysis showed that interactions with the neuroactive ligand-receptor pathways played a major role, providing a scientific basis for further clarifying the mechanism of action for Compound Anshen essential oil in the treatment of insomnia.

## Figures and Tables

**Table 1:** Chemical composition and the topological attributes of Compound Anshen essential oil.

No.	Composition	Degree	No.	Composition	Degree
1	2-Thujene	2	12	(-)-*α*-Terpineol	17
2	*α*-Pinene	9	13	D-Citronellol	1
3	*β*-Phellandrene	3	14	Linalyl anthranilate	11
4	*β*-Pinene	12	15	Lavandulyl acetate	1
5	3-Octanone	5	16	Caryophyllene	22
6	p-Cymene	6	17	(-)-*β*-Famesene	14
7	D-Limonene	12	18	*β*-Santalene	2
8	Ocimene	1	19	*α*-Santalol	1
9	*γ*-Terpinene	6	20	Nonadecane	3
10	Linalool	18	21	Dibutyl phthalate	29
11	(-)-4-Terpineol	16	22	Eicosane	1

**Table 2:** Information on potential targets and the topological attributes.

No.	Gene name	Protein name	UniProt ID	Degree
1	GABRA2	Gamma-aminobutyric acid receptor subunit alpha-2	P47869	10
2	GABRA1	Gamma-aminobutyric acid receptor subunit alpha-1	P14867	15
3	GABRA5	Gamma-aminobutyric acid receptor subunit alpha-5	P31644	6
4	GABRA6	Gamma-aminobutyric acid receptor subunit alpha-6	Q16445	9
5	ESR1	Estrogen receptor	P03372	12
6	NR1I2	Nuclear receptor subfamily 1 group I member 2	O75469	13
7	CYP19A1	Aromatase	P11511	9
8	NR1H4	Bile acid receptor	Q96RI1	9
9	NR1I3	Nuclear receptor subfamily 1 group I member 3	Q14994	12
10	CHRM3	Muscarinic acetylcholine receptor M3	P20309	8
11	CHRM2	Muscarinic acetylcholine receptor M2	P08172	10
12	CHRM1	Muscarinic acetylcholine receptor M1	P11229	9
13	PTGS1	Prostaglandin G/H synthase 1	P23219	7
14	SLC6A2	Sodium-dependent noradrenaline transporter	P23975	8
15	GABRA3	Gamma-aminobutyric acid receptor subunit alpha-3	P34903	6
16	CYP3A4	Cytochrome P450 3A4	P08684	3
17	HCRTR1	Orexin receptor type 1	O43613	2
18	DRD2	D(2) dopamine receptor	P14416	2
19	ADRB2	Beta-2 adrenergic receptor	P07550	5
20	OPRD1	Delta-type opioid receptor	P41143	2
21	OPRM1	Mu-type opioid receptor	P35372	2
22	DRD1	D(1A) dopamine receptor	P21728	2
23	SMPD1	Sphingomyelin phosphodiesterase	P17405	2
24	TRPV1	Transient receptor potential cation channel subfamily V member 1	Q8NER1	2
25	ADRA1B	Alpha-1B adrenergic receptor	P35368	3
26	KCNH2	Potassium voltage-gated channel subfamily H member 2	Q12809	2
27	HTR3A	5-hydroxytryptamine receptor 3A	P46098	1
28	CALM1	Calmodulin-1	P0DP23	1
29	SLC6A3	Sodium-dependent dopamine transporter	Q01959	4
30	ADRA1D	Alpha-1D adrenergic receptor	P25100	1
31	ADRA1A	Alpha-1A adrenergic receptor	P35348	4
32	UGT1A1	UDP-glucuronosyltransferase 1-1	P22309	2
33	SLC6A4	Sodium-dependent serotonin transporter	P31645	2
34	CA2	Carbonic anhydrase 2	P00918	1
35	GABRA4	Gamma-aminobutyric acid receptor subunit alpha-4	P48169	1
36	CHRNA7	Neuronal acetylcholine receptor subunit alpha-7	P36544	2
37	HTR2A	5-hydroxytryptamine receptor 2A	P28223	1
38	CHRM4	Muscarinic acetylcholine receptor M4	P08173	4
39	CHRM5	Muscarinic acetylcholine receptor M5	P08912	1
